# Exploring Future Signals of COVID-19 and Response to Information Diffusion Using Social Media Big Data

**DOI:** 10.3390/ijerph20095753

**Published:** 2023-05-08

**Authors:** Juyoung Song, Dal-Lae Jin, Tae Min Song, Sang Ho Lee

**Affiliations:** 1 Criminal Justice, Pennsylvania State University, Schuylkill, PA 17972, USA; 2 Department of Public Health, Graduate School of Korea University & Transdisciplinary Major in Learning Health Systems, Korea University, Seoul 02841, Republic of Korea; 3 School of Industry and Environment, Gachon University, Seoul 13120, Republic of Korea; 4 CEO for HealthMax Co., Ltd., Seoul 06078, Republic of Korea

**Keywords:** future signals, COVID-19, information diffusion, social big data

## Abstract

COVID-19 is a respiratory infectious disease that first reported in Wuhan, China, in December 2019. With COVID-19 spreading to patients worldwide, the WHO declared it a pandemic on 11 March 2020. This study collected 1,746,347 tweets from the Korean-language version of Twitter between February and May 2020 to explore future signals of COVID-19 and present response strategies for information diffusion. To explore future signals, we analyzed the term frequency and document frequency of key factors occurring in the tweets, analyzing the degree of visibility and degree of diffusion. Depression, digestive symptoms, inspection, diagnosis kits, and stay home obesity had high frequencies. The increase in the degree of visibility was higher than the median value, indicating that the signal became stronger with time. The degree of visibility of the mean word frequency was high for disinfectant, healthcare, and mask. However, the increase in the degree of visibility was lower than the median value, indicating that the signal grew weaker with time. Infodemic had a higher degree of diffusion mean word frequency. However, the mean degree of diffusion increase rate was lower than the median value, indicating that the signal grew weaker over time. As the general flow of signal progression is latent signal → weak signal → strong signal → strong signal with lower increase rate, it is necessary to obtain active response strategies for stay home, inspection, obesity, digestive symptoms, online shopping, and asymptomatic.

## 1. Introduction

The outbreak of the novel coronavirus disease (COVID-19), which causes various respiratory symptoms, became a global issue in January 2020 [1]. Although COVID-19 infections began in Wuhan, China, in December 2019 [2,3], Korea saw its first confirmed patient on 20 January 2020, and group infections emerged due to exposure as confirmed patients moved about. The WHO reported that the majority of people infected with COVID-19 experience mild to moderate respiratory symptoms, and the elderly and people suffering from cardiovascular conditions, diabetes, and chronic respiratory diseases are more likely to develop serious illness [4]. Common COVID-19 symptoms include fever, dry cough, and fatigue, and on rarer occasions, people may experience body aches, sore throat, diarrhea, conjunctivitis, headache, loss of taste of smell, skin rash, discoloration of fingers or toes, and muscle pain. More serious symptoms include difficulty breathing, chest pain and tightness, and speech or movement disorders [3,4,5,6,7]. Key comorbid symptoms of COVID-19 patients include high blood pressure, diabetes, shortness of breath, and vomiting [8,9,10].

It is currently unclear whether complications from the COVID-19 pandemic affect the risk and severity of infection with COVID-19. In Korea, a retrospective controlled study was conducted on 219,961 people aged 18 years or older who were charged for medical expenses for COVID-19 testing by 15 May 2020. The proportion was high at 59.5% (4027 people), and in terms of COVID severity, it was found that older men had a higher proportion than the non-severe group [8].

In [9], Danish patients diagnosed with COVID-19 after the outbreak of COVID-19 until 3 May 2020 were identified to calculate the exposure of interest to comorbidities 10 years before the outbreak of COVID-19. A total of 4480 patients were diagnosed with COVID-19, of which 65% had a Charlson Comorbidity Index Score (CCIS) of 0, 24.8% CCIS 1–2, 6.4% CCIS 3–4, and 3.8% had 4 or more. Overall, 17.8% had serious severe outcomes, of which 9.3% reported death. In the CCIS 0 group, only 9.1% had a serious outcome and 77 died. In the CCIS 1–2 group, 29.7% had a serious outcome and 17.7% died, in the CCIS 3–4 group, 42.7% had a serious outcome and 29.5% died, and in the CCIS 4 or more group, 473 % had serious consequences and 34.3% died [9].

Ref. [10] evaluated the risk of developing serious side effects by stratifying the co-morbidity status of patients with COVID-19. A total of 1590 inpatients were analyzed. Their average female was 48.9 years old, and the number of females was 42.7%, which was 686, and 16% of the population was severe. In addition, 399 patients (25.1%) were found to have at least one comorbidity, with hypertension accounting for 16.9% and diabetes accounting for 8.2% as the most common comorbidity. Patients with one or more comorbidities had a mean age of 44.8 years and were more likely to have shortness of breath, nausea and vomiting [10].

The most significant threats of the spread of the pandemic are potentially high mortality rates and high economic costs [11]. While humankind has seen various infectious diseases such as dengue fever, malaria, influenza, and HIV/AIDS, it is difficult to build appropriate epidemiological models for these diseases, and some scientists consider the spread of the pandemic a complex network of prediction and modeling [12].

As COVID-19 spreads worldwide, it is important to understand epidemiological models of its diffusion, mortality, and recovery [11,13].

In response to the COVID-19 pandemic, most countries adopted social distancing, and the “social distancing” policy was used to limit the spread of COVID-19 in the long term. It is a policy that aims to prevent infection and contain its spread while maintaining daily life, economic, and social activities.

Governments worldwide are continually working to treat infected patients, educate people about social distancing, test potential treatments and vaccines, and reduce the spread of infections. Although governments and organizations face economic and social threats to safely and gradually reopen the society, there is a lack of scientific evidence about how to do this [14]. Since Korea experienced the Middle East Respiratory Syndrome (MERS) in 2015, the Korea Centers for Disease Control and Prevention and many hospitals in Korea have been preparing for an infectious outbreak. When an explosive outbreak occurred in Daegu and Gyeongsangbuk-do in February 2020, Korea successfully conducted group examinations to identify mild or asymptomatic patients to prevent the spread of COVID-19. It also designed and implemented drive-thru testing centers to stop and overcome the spread of COVID-19 [15]. Furthermore, it chose social distancing, a type of nonpharmaceutical intervention used to slow the spread of respiratory viruses [16,17,18], as a basic measure to protect against COVID-19, preventing further spread of infections during this period [15,19].

Social media channels such as Facebook, Twitter, and WeChat [20] have become important sources of health information for global users. With the spread of COVID-19, both correct and false information have spread across social media. The diffusion of misinformation can lead to the masking of healthy behavior and increasing the spread of the virus, leading to negative physical and mental outcomes for individuals (stress, depression, etc.) [21]. Accurate predictions are important to slow and prevent infections [22]. A health informatics monitoring system was developed that utilizes social media information for the early prediction and monitoring of diseases, and the availability of social network media data, particularly that of Twitter, allowed for real-time monitoring of potential outbreaks, including follow-up measures, immediate analyses, and feedback [23]. One of the most important factors in the progression of a pandemic is the rate of its spread and what measures can be taken to slow the spread. Social media has emerged as an important medium that identifies and explains emergency situations for both governments and citizens [24]. In times of crisis, many countries have utilized social media for communication and crisis management [25]. A study by Wukich [26] explained that US state agencies use official social media accounts to disclose different stages of disasters and provide information on early warning and guidance, government measures, and correction of misinformation. Yum [27] conducted an analysis of the influence of public key players on Twitter regarding coronavirus in the United States and found that Barack Obama played an important role in social media, while his centrality was relatively low compared to his followers. Donald Trump, who had fewer followers than Barack Obama, had the greatest influence on social media. Jurgens and Helsloot [28] found that social media platforms allow people to search for and share trusted information, and that social media can improve people’s ability to understand the current situation and cooperatively solve problems due to its connective capabilities.

Najmul Islam et al. [29] analyzed the effects of sharing unconfirmed information as news and personal experiences during an outbreak of an epidemic, and found that those who were engaged in marketing and entertainment and those who lack self-regulation were more likely to share unconfirmed information. A study on the risk of information diffusion related to MERS by Song et al. [30] found that negative information was being spread on Twitter, and positive information was being spread on news channels. Cuan-Baltazar et al. [31] analyzed information quality by collecting data using the keywords “coronavirus” and “Wuhan” through the Google search engine from the outbreak of COVID-10 until 6 February 2020, and found that 70.0% of the websites had low-quality information. Furthermore, there were significant health risks associated with health information online. Governments should develop strategies to regulate health information on the Internet, because wrong information, false news, and misinformation about COVID-19 can create difficulties for public health guidelines and prevention. This is because people are hungry for information and are willing to trust confusing information and spread such information [32].

Today, AI technologies and tools are playing a key role in all aspects of responding to the COVID-19 crisis. As the coronavirus becomes a global pandemic, AI can be used to help manage all stages of the pandemic crisis and its aftermath (detection, prevention, response, and recovery) across society, including policy and health care [33].

Hiltunen (2008) explained the weak signal as a three-dimensional future signal space such as signal, issue, and interpretation using the concept of future sign [34]. Yoon (2012) created keyword portfolios of KEM (Keyword Emergence Map) and KIM (Keyword Issue Map) using word frequency, document frequency, and occurrence frequency increase rate, and selected weak signals using them. KEM can calculate DoV (Degree of Visibility) by showing visibility, and KIM can calculate DoD (Degree of Diffusion) by showing the degree of diffusion [35].

For this study, we examined tweets related to COVID-19 on Twitter, to explore the future signals of COVID-19 in Korea and prepare responses for information diffusion.

## 2. Materials and Methods

### 2.1. Data

The collection of tweets utilized the Twitter Application Programming Interface and included users whose last tweet was 365 days ago or less. Using the user information in the Twitter pool, a crawler was used to collect data from original tweets (not retweets) made from 2 February 2020 to 31 May 2020 about relevant topics related to COVID-19. For the natural language processing of the collected tweets (unstructured text documents), morpheme analysis was conducted using methodologies such as head–tail classification, left–right and right–left analysis, and syllable unit analysis. The data refining was performed by keyword extraction through morpheme analysis of the collected documents, and the documents relating to advertisement posts were filtered and excluded.

A total of 1,746,347 posts related to COVID-19 were found, and among them, 373,562 posts based on ontology classification and 5 subjects (symptom, response, prevention, issue, industry) were utilized as targets for the analysis of future signals related to COVID-19.

This study was conducted after receiving the approval of the Institutional Review Board (2-1040781-A-N-012020056HR). The participants of this study cannot be personally identified from the data, ensuring their confidentiality and anonymity.

### 2.2. Text Mining

Tweets are unstructured text documents with a limit of 140 characters. Ontology is required to extract meaningful keywords from them, extract COVID-19 concepts, and show the relationships between concepts. Ontology, a computer-interpretable knowledge model that formalizes and represents shared concepts of a topic of interest [36], is required to classify and process large-scale unstructured text documents, which can then be analyzed through existing research methodologies. We developed an ontology by analyzing papers and press releases on related topics of COVID-19, and conducted text mining of COVID-19-related tweets. The ontology classification of COVID-19 in this study consisted of 5 subjects: symptoms, response, prevention, issue, and industry. Symptoms were classified into 9 factors: positive case, negative case, fever, respiratory, cold, sore throat, digestive symptoms, and depression. Response was classified into 6 factors: inspection, treatment, quarantine, diagnosis kit, government response, and school closed. Prevention was classified into 5 factors: immunity food, health care, hand cleaner, disinfectant, and mask. Issue was classified into 4 factors: no entry, infodemic, obesity, and stay home. Industry was classified into 5 factors: regional economy, tourism industry, culture industry, online shopping, and airline.

### 2.3. Future Signal Analysis

To explore future signals by analyzing posts on Twitter, it was necessary to calculate the term frequency and document frequency of the relevant factors therein. Term frequency is the total number of times a word is used in all relevant documents. Document frequency indicates the number of documents in which a specific word appears. The study used “R” program to conduct the analysis.

We then calculated the degree of visibility (*DoV*), and the degree of diffusion (*DoD*) [37]. The following equations are used to calculate *DoV* and *DoD*. The symbols in the equations signify the following:

$${\mathrm{DoV}}_{ij}=(TF_{ij}/NN_{j})\;\times \;\{1\;-\;\mathrm{tw}\times \;(n-j)\}$$

$${\mathrm{DoD}}_{ij}=(DF_{ij}/NN_{j})\;\times \;\{1\;-\;\mathrm{tw}\times \;(n-j)\}$$

*NN*: total number of documents;
*TF*: term frequency;
*DF*: document frequency;
*tw*: time weight (time weight of 0.05 was applied);
*n*: entire time segment;
*j*: time point;
“*{1 − tw × (n − j)}*” is a function that weakens its influence as time passes, thus determining the scale of the time weight [37]. Keyword emergence map (KEM) and Keyword Issue Map (KIM) were examined by visualizing the frequency of emergence and the time-weighted rate of the increase in topics [35,37].

## 3. Results

As shown in Table 1, this study calculated the mean DoV increases and mean term frequency for each of the major factors of COVID-19 topics and found that the median value of DoV increase was 0.145, indicating that the mention of major factors increased. Stay home, inspection, obesity, depression, diagnosis kit, digestive symptoms, asymptomatic, online shopping, culture industry, negative case, regional economy, quarantine, government response, and treatment showed high frequencies, and the DoV increased to be higher than the median value, indicating that their frequency grew stronger over time. The mean term frequencies of disinfectant, cold, health care, mask, positive case, infodemic, tourism industry, and airline were high, but the DoV increase rates were lower than the median, indicating that their use grew weaker over time.

As shown in Table 2, DoD showed similar trends as DoV. However, the median value of DoD increase was 0.101, indicating that the diffusion of the major factors by each COVID-19 subject increased. Diagnosis kit, negative case, culture industry, regional economy, quarantine, government response, and treatment showed high frequencies, with DoD increasing to be higher than the median, indicating that their signals grew stronger over time. The mean term frequencies of disinfectant, cold, infodemic, hand cleaner, and positive case were high; their DoD increases were lower than the median, indicating that their signals grew weaker over time.

To explore future signals, we set the mean term frequency of DoV and mean document frequency of DoD as the *x*-axis and the mean increases in DoV and DoD as the *y*-axis. Then, we divided the median values of each into quadrants—the keywords in Quadrant 2 have weak signals, the keywords in Quadrant 1 have strong signals, the keywords in Quadrant 3 have latent signals, and the keywords in Quadrant 4 have strong signals but with slow increase rates.

**Figure 1:**
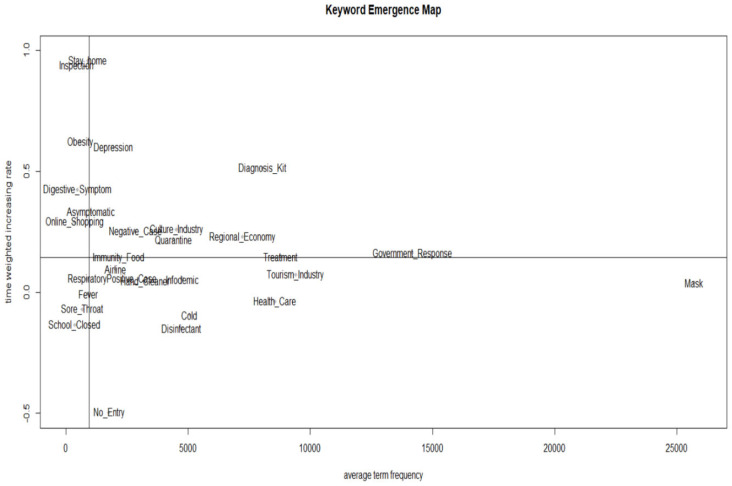
KEM (Keyword Emergence Map) of main factors related to COVID-19.

**Figure 2:**
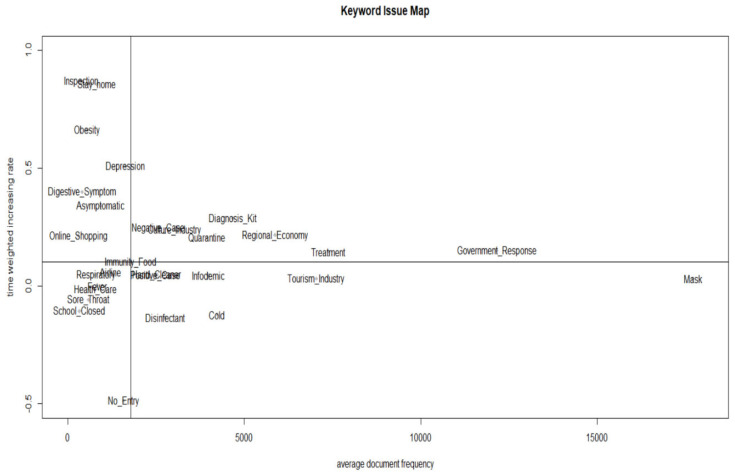
KIM (Keyword Issue Map) of main factors related to COVID-19.

As shown in Figure 1 and Figure 2, and Table 3, depression was found to be a strong signal in the KEM, but a weak signal in the KIM in terms of the main factors of COVID-19. This indicated that the influence of the depression factor decreased. The strong signals common to both KEM and KIM (Quadrant 1) included culture industry, quarantine, negative case, diagnosis kit, regional economy, treatment, and government response. Weak signals (Quadrant 2) included stay home, inspection, obesity, digestive symptoms, online shopping, and asymptomatic. Latent signals (Quadrant 3) included fever, sore throat, school closed, respiratory, immunity food, and no entry. Strong signals with slow increase rates in (Quadrant 4) included infodemic, cold, disinfectant, hand cleaner, tourism industry, and mask.

## 4. Discussion

The COVID-19 pandemic has caused serious problems worldwide and has led to rapid increases in rumors and misinformation about the causes, results, prevention, and treatment of the disease. Collecting and analyzing big data related to COVID-19 on social media could lead to effective responses to COVID-19 by providing correct knowledge and information. Social media data analysis can be used to track incorrect information and unconfirmed rumors and can help in understanding the fear associated with the disease. Furthermore, it can be used for social mobilization, health promotion, and communication with the public about public health interventions.

This study examined 1,746,347 posts on Twitter related to COVID-19 to explore the future signals of COVID-19 and responses to information diffusion. We analyzed the term frequency, document frequency of the key factors, importance, and degree of diffusion to explore future signals.

Depression, digestive symptoms, asymptomatic keywords relating to symptoms, inspection and diagnosis kit relating to response, and stay home and obesity relating to issues showed a high frequency. The results indicate that their signals strengthened over time. Therefore, it is necessary to actively advertise the symptoms of COVID-19 and engage in active testing for early detection of confirmed patients, ensuring that regional outbreaks do not occur. Furthermore, it is necessary to take preemptive measures against asymptomatic patients by understanding the degree of the COVID-19 outbreak by constructing a community sample monitoring system to ensure that outbreaks are not driven by asymptomatic patients.

The DoV mean term frequency was high for disinfectant, health care, and mask, but the signals were growing weaker over time. Reemergence of COVID-19 outbreaks are a concern, given people’s fatigue of wearing masks long term, and the avoidance of mask wearing during the summertime. Therefore, it is necessary to continuously reinforce public communication on prevention measures and mask wearing.

The issue of infodemic had a high DoD term frequency, but the results indicated that the signal weakened over time. The diffusion of misinformation related to COVID-19 is difficult to correct and may lead to excessive fear or psychological issues such as depression and anxiety. Therefore, management of the infodemic issue and fact checking at the governmental level is necessary to provide reliable information.

Stay home and obesity showed high increasing rates, indicating that these keywords may grow into strong signals. As such, active countermeasures are needed for these issues. Regional outbreaks of COVID-19 may lead to obesity as people spend time at home, so it is necessary to provide people with information on exercise methods that can be carried out at home. Moreover, the prevalence of obesity in COVID-19 patients admitted to the intensive care unit was disproportionately high compared to the general public [38], and it has been reported that when obese people are exposed to COVID-19, they are more likely to be hospitalized [39]. Therefore, it is necessary to intervene to improve lifestyles and obesity to reduce the risk of COVID-19, and it is necessary for governments to strengthen social distancing measures for severely obese individuals. Furthermore, the government should provide clear guidelines for risk management for infections in obese patients.

Finally, depression as a symptom was found to be a strong signal in the KEM but a weak signal in the KIM. This indicates that the influence of depression as a symptom and a major factor of COVID-19 continues to decrease, requiring subject-specific psychological treatment and stress management at the government level. Furthermore, as the COVID-19 crisis continues, there are more people stricken with depression from the fear of infections as well as with the overwhelming stress of unemployment, death, and social isolation. The importance of improving the psychological quarantine system should be emphasized as anxiety and fear associated with COVID-19 spreads.

The policy suggestions and limitations of this study are as follows: First, the typical flow of a signal progresses from a latent signal (low frequency and increasing rate) → weak signal (low frequency and high increasing rate) → strong signal (high frequency and increasing rate) → high signal with low increasing rate (high frequency and low increasing rate). When a signal has a low frequency and an increasing rate, it can be interpreted as a latent signal, as it has not emerged as an issue. However, weak signals with low frequency and a high increasing rate are likely to become issues in the future. Furthermore, strong signals with high frequency and an increasing rate are keywords that have already emerged as issues. Therefore, the weak signals of stay home, inspection, obesity, digestive symptoms, online shopping, and asymptomatic can become strong signals within a short period of time, requiring countermeasures.

Second, COVID-19 terms on Twitter may include colloquial speech and slang often found on Twitter as well as the professional terms classified under the theoretical background of ontology; as such, the COVID-19 ontology should be continuously revised and supplemented.

Third, the ontology developed in this study can contribute to the development of a chatterbot that can immediately predict the risk of COVID-19 by learning unstructured data.

Fourth, the COVID-19-related terms used in the subject analysis of this study may differ in definition compared to the COVID-19 terms in existing offline surveys.

In conclusion, as the second wave of the pandemic approaches, there are difficulties associated with developing a COVID-19 vaccine, and even if one is developed, it is likely that its development will happen after the second wave. Until the effectiveness of a COVID-19 vaccine has been proven and its distribution is sufficient for people around the world to be vaccinated and develop immunities, the world has no choice but to prevent COVID-19 through personal quarantines, social distancing, mask wearing, and strict hygiene management. Furthermore, it is necessary for people to strengthen their own individual immunity to reduce the risk of COVID-19 infection. Finally, it is necessary to strengthen the monitoring mechanism through preemptive measures such as rapid diagnosis and the isolation of confirmed patients.

## Figures and Tables

**Table 1:** DoV mean increase rate and mean term frequency for main factors related to COVID-19.

Keyword	DoV				Mean Increase Rate	Mean Term Frequency
	2020 February	2020 March	2020 April	2020 May		
Mask	27,771	44,034	17,173	13,812	0.041	25,698
Government Response	12,714	20,865	13,540	9572	0.162	14,173
Tourism Industry	13,536	10,974	6573	6529	0.072	9403
Treatment	7302	13,276	9705	4843	0.150	8782
Health Care	11,051	11,884	6653	4623	−0.038	8553
Diagnosis Kit	3945	15,485	9801	2945	0.513	8044
Regional Economy	5944	10,024	7704	5223	0.231	7224
Cold	8294	6408	3017	2537	−0.093	5064
Infodemic	5107	7627	3576	2804	0.056	4779
Disinfectant	6557	6689	3764	1896	−0.148	4727
Culture Industry	4563	5396	4076	4057	0.260	4523
Quarantine	3382	6178	5897	2150	0.219	4402
Hand Cleaner	3919	4241	2817	2061	0.045	3260
	3204	4697	1486	2027	0.252	2854
	2714	4387	2169	1500	0.055	2693
Immunity Food	2274	2877	1927	1601	0.145	2170
Airline	2102	2689	2091	1259	0.097	2035
Depression	1064	2618	2054	2064	0.600	1950
Negative Case	3296	2888	693	190	−0.497	1767
Positive Case	739	1470	1129	824	0.333	1041
Fever	1256	1260	639	550	−0.004	926
Stay home	348	958	1778	516	0.958	900
Respiratory	1037	1139	769	563	0.056	877
Sore Throat	1043	989	342	325	−0.070	675
Obesity	369	662	617	739	0.623	597
Digestive Symptom	379	722	347	449	0.425	474
Inspection	153	617	586	412	0.937	442
School Closed	555	621	160	135	−0.135	368
Online Shopping	530	340	231	326	0.292	357
Median					0.145	2693

**Table 2:** DoD mean increase rate and mean document frequency for main factors related to COVID-19.

Keyword	DoD				Mean Increase Rate	Mean Document Frequency
	2020 February	2020 March	2020 April	2020 May		
Mask	19,399	27,900	13,314	10,225	0.031	17,710
Government Response	10,880	17,898	11,728	8115	0.150	12,155
Treatment	6150	11,124	8079	4194	0.146	7387
Tourism Industry	10,477	8182	4847	4679	0.031	7046
Regional Economy	4891	8227	6082	4307	0.216	5877
Diagnosis Kit	2910	8835	5211	1774	0.287	4683
Cold	6898	5497	2509	2038	−0.121	4236
Infodemic	4266	6265	3100	2357	0.045	3997
Quarantine	3010	5567	5226	1944	0.206	3937
Culture Industry	3090	3654	2635	2702	0.239	3020
Disinfectant	3760	3980	2101	1206	−0.133	2762
Negative Case	2914	4175	1348	1856	0.246	2573
Hand Cleaner	2928	3285	2285	1616	0.048	2529
Positive Case	2563	4060	1951	1395	0.044	2492
Immunity Food	2001	2367	1504	1281	0.101	1788
Depression	990	2315	1614	1628	0.510	1637
No Entry	2921	2587	648	181	−0.488	1584
Airline	1289	1583	1276	700	0.060	1212
Asymptomatic	664	1313	1010	778	0.340	941
Fever	1117	1137	576	511	0.002	835
Stay home	337	924	1596	475	0.857	833
Respiratory	957	1028	695	522	0.047	801
Health Care	1060	982	615	469	−0.014	782
Sore Throat	887	866	304	295	−0.058	588
Obesity	321	604	574	716	0.662	554
Digestive Symptom	341	629	295	385	0.400	413
Inspection	141	508	523	368	0.871	385
School Closed	495	572	142	128	−0.107	334
Online Shopping	424	314	207	263	0.214	302
Median					0.101	1788

**Table 3:** Future signals of main factors related to COVID-19.

Future Signal	Latent Signal	Weak Signal	Strong Signal	Strong But Low Increasing Signal
KEM	Fever, Sore Throat, School Closed, Positive Case, Respiratory, Immunity Food, No Entry	Stay home, Inspection, Obesity, Digestive Symptom, Online Shopping, Asymptomatic	Depression, Culture Industry, Quarantine, Negative Case, Diagnosis Kit, Regional Economy, Treatment, Government Response	Airline, Infodemic, Cold, Disinfectant, Hand Cleaner Tourism Industry, Health Care, Mask
KIM	Fever, Sore Throat, School Closed, Respiratory, Immunity Food, No Entry, Airline, Health Care	Stay home, Inspection, Obesity, Digestive Symptom, Online Shopping, Asymptomatic, Depression	Culture Industry, Quarantine, Negative Case, Diagnosis Kit, Regional Economy, Treatment, Government Response	Infodemic, Cold, Disinfectant, Hand Cleaner, Tourism Industry, Mask, Positive Case,
Common signal	Fever, Sore Throat, School Closed, Respiratory, Immunity Food, No Entry	Stay home, Inspection, Obesity, Digestive Symptom, Online Shopping, Asymptomatic	Culture Industry, Quarantine, Negative Case, Diagnosis Kit, Regional Economy, Treatment, Government Response	Infodemic, Cold, Disinfectant, Hand Cleaner, Tourism Industry, Mask

## Data Availability

Not Applicable.
